# “90/90” Plating of proximal humerus fracture—a technical note

**DOI:** 10.1186/s13018-019-1083-3

**Published:** 2019-02-11

**Authors:** John Tristan Cassidy, Eamonn Coveney, Diarmoud Molony

**Affiliations:** Tallaght University Hospital, Dublin, Ireland

**Keywords:** Proximal humerus fracture, Medial comminution, Varus deformity, Open reduction internal fixation

## Abstract

**Introduction:**

While locking plates have markedly improved fixation of proximal humerus fractures, a cohort of fractures remains difficult to treat. This cohort has been identified as fractures with marked medial comminution and varus deformity. Loss of reduction and fixation failure are the most frequently reported complications for this cohort. We report the use of an orthogonal 1/3 tubular plate to augment the proximal humerus locking plate.

**Methods:**

The subject underwent osteosynthesis for a four-part proximal humerus fracture with medial comminution. Fixation was performed within 24 h of injury. Standard deltopectoral approach exposed the fracture. Sutures were sited to control the tuberosities and cuff. Initial reduction was held with a K-wire and augmented with a three-hole 1/3 tubular plate. Proximal humerus locking plate was sited in standard fashion including locked medial support screws. Reduction was confirmed both clinically and with intra-operative radiography.

**Results:**

The technique provided satisfactory results. At 6 months, the fracture had fully united with no loss of reduction. At 1 year, the patient had excellent range of motion.

**Conclusion:**

The use of a 1/3 tubular plate to augment fixation of proximal humerus fractures with medial comminution may provide a simple, reproducible, and cost-effective method to decrease loss of reduction and subsequent malunion.

## Background

Locked plating systems allow surgeons greater control of both the fracture fragments and the rotator cuff for proximal humerus fractures. However, certain fracture configurations remain challenging, particularly with respect to maintaining reduction and avoiding malunion [1, 2]. While specifically designed locking plates provide excellent biomechanical control in the coronal plane, they have decreased ability to resist varus and extension forces [3]. This vulnerability is exacerbated intra-operatively, as extension deformities are more difficult to recognize [1, 2]. We present a novel use of an anterior 1/3 tubular plate paced orthogonally to a locking plate, with a 1-year radiological and functional follow-up.

## Case and surgical technique

**Fig. 1:**
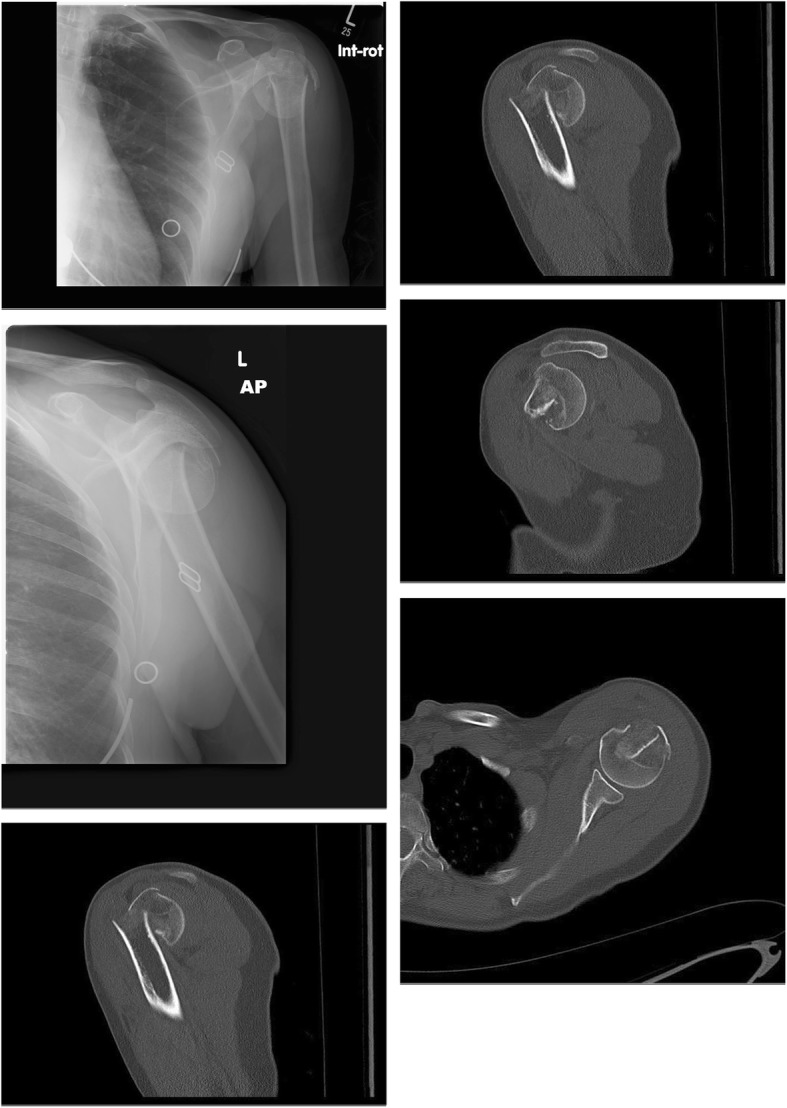
Imaging on presentation

A 60-year-old female required osteosynthesis of a proximal humerus fracture following a fall. Figure 1 displays plain x-rays and selected computed tomography slices at presentation. This is an anteromedial impaction fracture as classified by Furoria et al. This fracture configuration is associated comminution of the medial calcar with varus, flexion, and anteversion of the head as seen in Fig. 1 [4].

The patient was positioned beach chair with the affected arm controlled using a pneumatic limb positioner. Access was obtained via deltopectoral approach with biceps tenotomy (as is standard practice for the senior author). Sutures were placed in the tuberosities for both cuff control and fracture fragment manipulation. The varus impacted, apex-anterior extension deformity was reduced manually. This reduction was maintained using a K-wire and confirmed using an image intensifier. A three-hole 1/3 tubular plate was sited at the apex of correct the extension deformity. This was fixed with two uni-cortical non-locking screws. During fixation of the 1/3 tubular plate, the drill bit impacted the in situ K-wire and broke. The fragment was buried in the bone and considered unlikely to have a negative clinical impact. It was therefore left in situ.

**Fig. 2:**
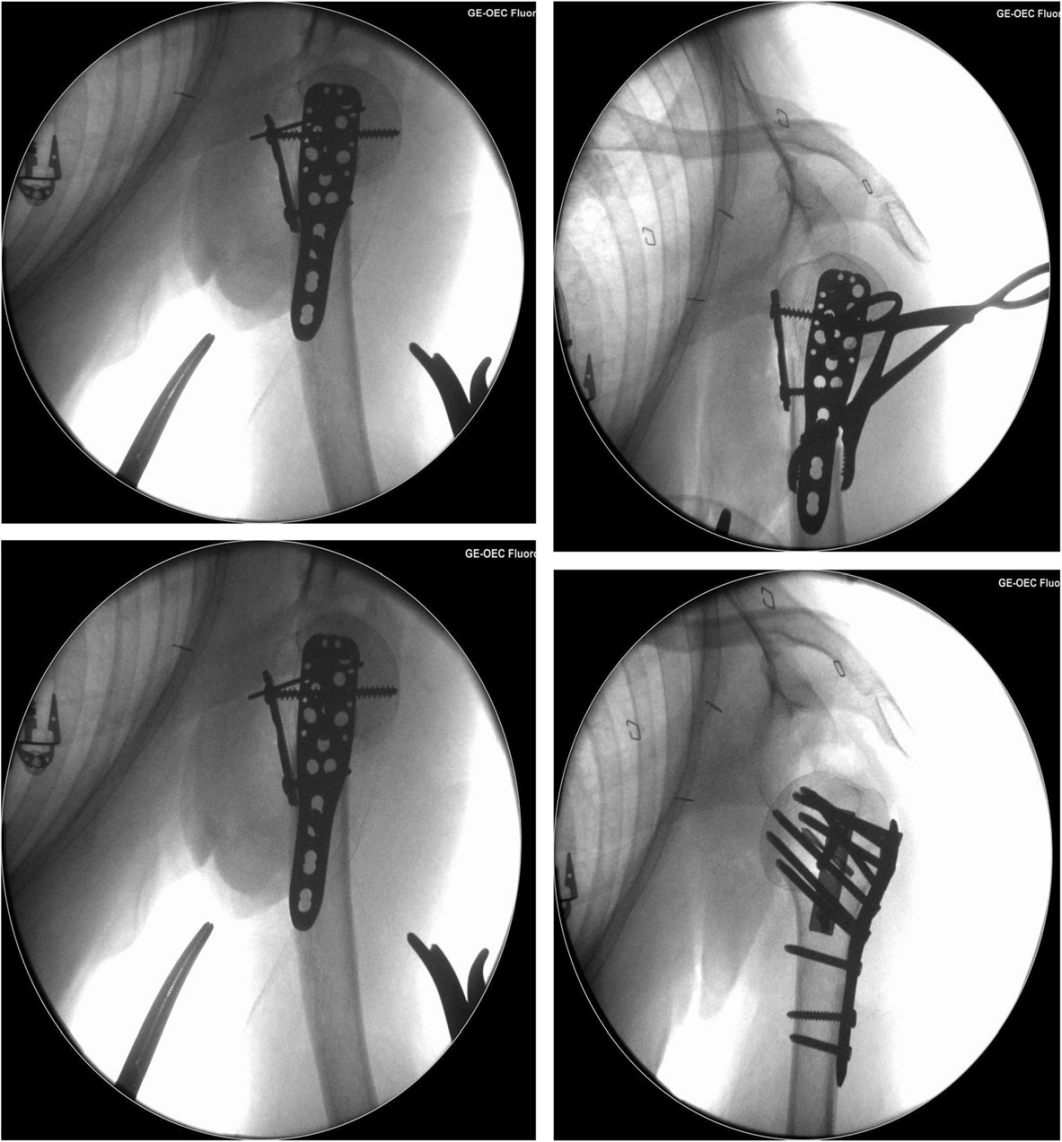
Intra-operative radiographs

Control of the fracture in the sagittal plane facilitated optimal positioning of a 3-hole proximal humerus locking plate without loss of reduction. A non-locking metaphyseal screw in the oblong hole allowed minor adjustments to final plate positioning prior to the insertion of seven proximal locking screws and two more (locking) metaphyseal screws. The tuberosities/cuff was reduced using the previously sited polyethylene sutures via the suture holes in the plate. These sutures were passed through the suture holes prior to locking screw insertion and tied after final plate positioning. Intra-operative x-rays are provided in Fig. 2. The tenotomized biceps tendon was sutured to the pectoral insertion completing a tenodesis, which is standard practice for the senior author.

## Rehabilitation, follow-up, and outcome

**Fig. 3:**
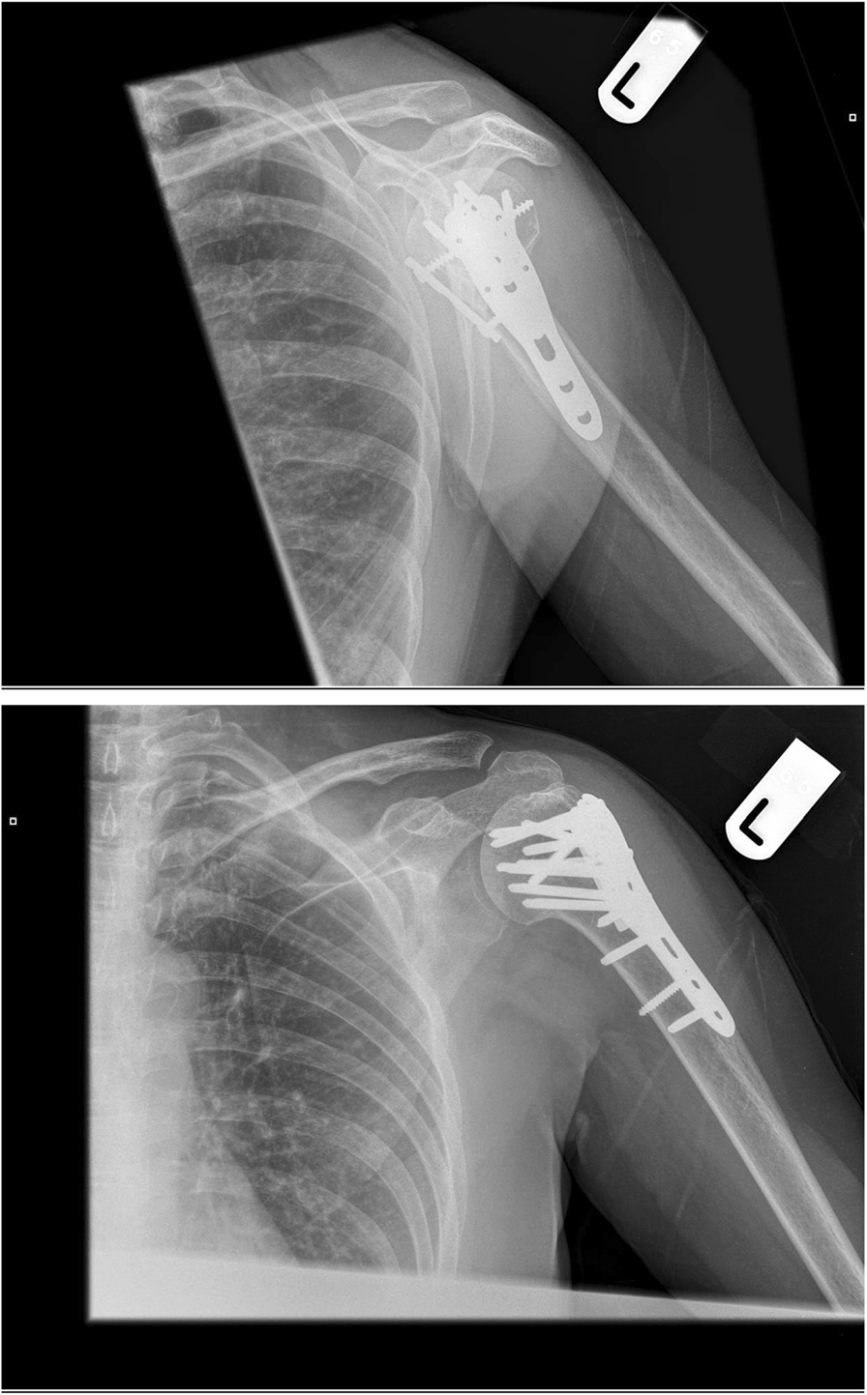
Radiographs at 1 year post-operatively

Following review by physiotherapy, the patient was discharged day 1 post-op. Range of motion was permitted immediately at the hand/wrist/elbow. Gentle active/active-assisted range of motion of the shoulder was commenced day 1 post-operatively. Subsequent reviews in the outpatients were at 2 weeks, 6 weeks, 2 months, 6 months and 1 year. The fracture was fully united at 6 months. X-rays at 1 year are provided in Fig. 3.

Active range of motion was assessed independently by the senior and second author at 1 year. Results for each assessor along with mean values are provided in Table 1. No cuff weakness was noted by either assessor.

**Table 1:** Active range of motion of shoulder at 1 year post-operatively^+^

	Assessor 1	Assessor 2	Mean
Forward flexion	150	130	140
Extension	30	20	25
Abduction	100	80	90
External rotation	0	0	0
Internal rotation*	L4	L5	L4/L5

^+^Expressed in degrees unless stated otherwise

*Expressed as the most proximal spinous process reached by the thumb

## Discussion

### Current fixation options and potential sources of improvement

Locking plates have become the standard of care in osteosythesis of proximal humerus fractures; however, high rates of complications and re-operation persist [5, 6]. A 2011 systematic review identified collapse into varus malunion as the most common complication following osteosythesis [5]. Reduction loss is a leading cause of revision surgery and ultimately results in poor outcomes [7].

Numerous variations of fixation methods have been described in and attempt to control these fractures, depending on the fracture configuration. These have included fixation with K-wires, use of bone cement, humeral nails, medialization with impaction of humeral shaft, use of allografts, and medial support screws [8–13].

Continued technical improvements and identification of risk factors for failure have been proposed as the two key elements for improved outcomes in varus impacted cohort [5]. Attempts to improve locking plate fixation in these patients have used three main strategies: (1) medial support screws, (2) cement augmentation, and (3) bone grafts [6]. While there is a paucity of biomechanical data to support the use of medial support screws, clinical studies suggest improved rates of reduction loss [14]. Calcium cement augmentation has been proposed to improve biomechanical properties and successful clinical results have been reported [9, 15]. Despite this however, it has failed to become established practice. Finally, the addition of intra-medullary fibular allograft has been shown to increase biomechanical robustness, but superior clinical results have not been demonstrated [6].

To date, low bone mineral density, age, and initial displacement have been associated with reduction loss. With specific respect to more technical aspects of fixation, medial comminution and insufficient medial support have also been associated with failure [7, 16]. Without medial support, i.e., bony contact, the stiffness of the construct is dramatically decreased and up to 43% of these fractures can be expected to subside into varus [17]. It is postulated this is because locking plates for proximal humerus fractures have decreased ability to resist varus and extension deforming forces [3].

### The importance of the sagittal plane

While the importance of coronal malalignment has long been acknowledged by the literature, it is only in more recent years that sagittal alignment has been incorporated into the decision-making process and thus featured in classification systems [18]. It has been suggested that failure to control sagittal malalignment may affect periosteal blood supply and thus fracture healing [18]. This is consistent with the observation that varus impacted fractures with extension deformity are associated with progressive displacement, particularly when managed non-operatively [19]. Robinson et al. recognized these fractures to be technically challenging and associated with high rates of fixation failure with the use of a single plate [20]. Robinson et al. went on to describe the use of sculpted femoral head allograft to maintain bony continuity and prevent apex anterior deformity.

### Role of the “90/90” 1/3 tubular technique

In this technical note, we suggest the use of a single three-hole 1/3 tubular plate to control apex anterior deformity. This avoids both the increased costs associated with bone cement and allograft. Furthermore, it negates the potential risks associated with using allograft material. The placement of the 1/3 tubular plate at 90° has the further advantage of the fixation construct now directly resisting the two most important deforming forces—varus (locking plate) and extension (1/3 tubular plate). Thus, the “90/90” technique’s main role will be in the fractures with apex anterior sagittal deformities. One potential disadvantage of this method is restricting internal rotation. In this case the 1/3 tubular plate was placed over the tendinous portion of subscaularis. However, the results in this patient are comparable with the limited reports of internal rotation outcomes post-osteosythesis available in the literature [21, 22].

### Rationale for the proposed technique

By examining a cohort of 67 consecutive patients, Krappinger et al. identified age, local bone mineral density, initial displacement/angulation, and successful reduction as key factors for successful fracture healing [16]. However, half of these patients were managed by percutaneous fixation rather than open reduction with internal fixation. In 2015, Jung et al. reported bone density, varus displacement, medial comminution, and insufficient medial support to be independent risk factors for loss of reduction [7]. In our case, the use of the 1/3 tubular plate allowed better control of the bony fragments in the context of decreased bone mineral density as well as anatomical reduction. This is consistent with the biomechanical study performed by Schliemann et al. whereby anteriorly directed head screws augmented with bone cement were demonstrated to significantly decrease movement at the bone-implant interface [23].

The study by Schliemann et al. was the first to recognize the potential importance of the rigidity of the link between the plate and the humeral head [23]. The concept of using two orthogonal plates to create a more rigid construct is well recognized in elbows; however, it has not been examined in proximal humerus fractures [24]. Previous authors have successfully used two orthogonal 1/3 tubular plates prior to the introduction of locking plates, but this technique was overshadowed by the success of the proximal humerus locking plate [25]. Given a cohort with a high failure rate with locking plates has been identified, the authors propose this option should be re-examined but with a difference—partnering locking plates with an orthogonal 1/3 tubular plate. While there are previous biomechanical studies comparing locking plates with 1/3 tubular plates, we could not find any biomechanical or clinical studies examining their use in combination [26].

### Other described methods of augmenting locking plate fixation

Neviaser et al. described using an endosteal implant to aid fixation [27]. In four cases, a 1/3 tubular plate was used instead of fibular graft. This plate was placed medially with the intramedullary canal and locking screws were passed through both the locking plate and the 1/3 tubular plate. Thus, this technique differs in two ways to what we present. Firstly, the implant is intramedullary, and secondly the plates are in line rather than orthogonal [27]. While elegant, potential disadvantages such as increased difficult performing arthroplasty have been identified by other authors [28]. One potential advantage of the technique we describe is similar augmentation of the locking plate with less challenging access to both plates in the event of a revision.

### Limitations of the 90/90 technique

Potential limitations of the 90/90 technique include compromising range of motion (secondary to metal impingement on cuff) and threatening the blood supply to the humeral head, specifically the anterior circumflex artery [29]. However, these complications were not noted with the previous two plate constructs using 1/3 tubular plates [25]. With respect to range of motion, perhaps counterintuitively, it is external rotation which has recovered most poorly.

Little is known about external rotation outcomes post fixation, an observation confirmed by the 2015 Cochrane on operative intervention for proximal humerus fractures. However, the authors noted no clinically significant differences with for external rotation with any forms of intervention; although, it is more widely reported for arthroplasty in fractures. One possible cause of restricted internal rotation may be tethering of subscapularis beneath the plate. However, given the 1/3 tubular plate is sited at the tendinous insertion, this is unlikely. The lack of vascular consequence is likely explained by the fact that the anterior circumflex artery is disrupted in approximately 80% of fractures which is markedly higher than clinical rates of osteonecrosis [28, 30]. Finally, it may be difficult to site the anterior plate without performing a tenotomy of the long head of the biceps; however, in the fracture cohort, the cosmetic impact of this is unlikely to affect patient satisfaction.

## Conclusion

This technical note outlines a potentially cost-effective method of maintaining reduction in a challenging cohort of proximal humeral fracture patients (apex anterior deformity). No additional surgical exposure is required, and the only additional equipment needed is the widely available, cost-effective 1/3 tubular plate. The technique is therefore easily reproducible. By increasing rigidity at the fracture site, the 90/90 plate technique potentially mitigates a proposed source of failure.
